# Novel therapies for resistant focal segmental glomerulosclerosis (FONT) phase II clinical trial: study design

**DOI:** 10.1186/1471-2369-12-8

**Published:** 2011-02-10

**Authors:** Howard Trachtman, Suzanne Vento, Debbie Gipson, Larysa Wickman, Jennifer Gassman, Melanie Joy, Virginia Savin, Michael Somers, Maury Pinsk, Tom Greene

**Affiliations:** 1 Cohen Children's Medical Center of New York, North Shore Long Island Jewish Health System, New Hyde Park, NY, USA; 2 Department of Pediatrics, University of Michigan, Ann Arbor, MI, USA; 3 The Cleveland Clinic, Cleveland, OH, USA; 4 University of North Carolina, Chapel Hill, NC, USA; 5 Kansas City VA Medical Center, Kansas City, MO, USA; 6 Boston Children's Hospital, Boston, MA, USA; 7 University of Alberta, Edmonton AB, Canada; 8 University of Utah, Salt Lake City, UT, USA

## Abstract

**Background:**

The lack of adequate randomized clinical trials (RCT) has hindered identification of new therapies that are safe and effective for patients with primary focal segmental glomerulosclerosis (FSGS), especially in patients who fail to respond to corticosteroids and immunosuppressive therapies. Recent basic science advances have led to development of alternative treatments that specifically target aberrant pathways of fibrosis which are relevant to disease progression in FSGS. There is a need for a flexible Phase II study design which will test such novel antifibrotic strategies in order to identify agents suitable for phase III testing.

**Methods/Design:**

The Novel Therapies for Resistant Focal Segmental Glomerulosclerosis (FONT) project is a multicenter Phase I/II RCT designed to investigate the potential efficacy of novel therapies for resistant FSGS. Adalimumab and galactose will be evaluated against conservative therapy consisting of the combination of lisinopril, losartan and atorvastatin. The sample size is defined to assure that if one of the treatments has a superior response rate compared to that of the other treatments, it will be selected with high probability for further evaluation. Comparison of primary and secondary endpoints in each study arm will enable a choice to be made of which treatments are worthy of further study in future Phase III RCT.

**Discussion:**

This report highlights the key features of the FONT II RCT including the two-step outcome analysis that will expedite achievement of the study objectives. The proposed phase II study design will help to identify promising agents for further testing while excluding ineffective agents. This staged approach can help to prevent large expenditures on unworthy therapeutic agents in the management of serious but rare kidney diseases

**Trial Registration:**

ClinicalTrials.gov, NCT00814255

## Background

The goal of therapy in proteinuric diseases such as primary FSGS is complete remission of proteinuria and preservation of renal function. However, this is rarely achieved in patients with FSGS that is resistant to standard treatment [1,2]. When corticosteroids and immunosuppressive therapy fail to induce remission in patients with primary FSGS, a number of agents are used as renoprotective therapy to delay progression of chronic kidney disease (CKD) to end stage kidney disease (ESKD). Angiotensin converting enzyme inhibitor (ACEi) and angiotensin II receptor blocker (ARB) are two such therapies that reduce proteinuria when used alone, with an additive effect when prescribed in combination [3-12]. Prescription of 3-hydroxy-3-methyl-glutaryl coenzyme A (HMG-CoA) reductase inhibitors in doses designed to treat dyslipidemia is also associated with stabilization of glomerular filtration rate (GFR) and improved kidney function in chronic non-diabetic nephropathies [13]. Combined use of an ACEi, an ARB, and an HMG-CoA reductase inhibitors represents optimal conservative medical therapy in patients with resistant FSGS and has been advocated as a standard renoprotective regimen [14-17].

Great strides have been made in understanding renal fibrosis. Tumor necrosis factor-α (TNF-α) is an inflammatory cytokine produced by a wide range of cells including macrophages and renal tubular epithelial cells. Several mechanisms for TNF-α-induced proteinuria in FSGS have been proposed including recruitment of leukocytes to the site of glomerular injury, induction of cytokines and growth factors, generation of oxygen radicals with increased glomerular endothelial cell permeability, cytotoxicity, and induction of apoptosis [18-21]. The potential for TNF-α antagonism to reduce proteinuria in resistant FSGS is based on the finding of elevated TNF-α levels in experimental models of the disease and in patients with FSGS, induction of proteinuria in animals by TNF-α from mononuclear cells taken from patients with FSGS, and reduction in proteinuria with a TNF-α antagonist in the angiotensin II-induced renal injury model and other models that resemble FSGS [22].

Published reports indicate that serum samples of nearly 50% of patients with primary steroid resistant FSGS have the capacity to increase the permeability of glomeruli to albumin, (P_alb_) during *in vitro *incubation and testing [23]. Standard conditions for these studies include incubation of glomeruli from normal rats with medium containing 2% vol/vol patient serum. A value of to ≥ 0.5 is defined as a positive test [24]. Addition of 10^-12 ^M galactose to the incubation medium containing patient serum completely prevents the increase in permeability. Removal of galactose by extensive dialysis of the medium restores P_alb _activity. Intravenous administration of galactose or chronic ingestion of galactose prior to obtaining serum markedly decreases P_alb _activity. Dialysis of sera obtained after galactose administration does not restore activity, suggesting that galactose enhances removal of a circulating permeability factor [25]. Savin and colleagues have postulated that increased hepatic clearance by galactose-binding proteins (galectins) may be responsible for removal of the permeability factor. There are case reports describing individual patients with FSGS who were given oral galactose for over 6 months and who demonstrated reduction in P_alb_, lowering of proteinuria, and stabilization of kidney function [26]. These findings raise the possibility that extended administration of galactose to lower P_alb _in patients with resistant FSGS may reduce proteinuria and delay progressive decline in kidney function [27].

The FONT trial (DK70341) is a combined Phase I/II project with an overall objective to identify promising new antifibrotic agents for further testing and distinguishing them from agents which are likely to be ineffective. A staged approach to drug evaluation is incorporated into the Phase II trial design to avoid large expenditures on unworthy and untested therapeutic agents for this serious disease.

## Methods/Design

### Study Design: General considerations

FONT II is a Phase II open-label RCT to choose which treatment or treatments are worthy of further study in future Phase III studies.

### Specific Aims

1. To evaluate two novel therapies for resistant FSGS - adalimumab, a human anti-TNF-α antibody, and galactose - compared to standard conservative therapy;

2. To identify one or more novel agents as candidates for evaluation in a future Phase III RCT; and

3. To create and sustain an infrastructure for the timely completion of RCTs in patients with rare glomerular diseases like primary FSGS.

### Primary and Secondary Outcomes

There will be a composite endpoint in which a patient will be classified as having a positive response if both of the following criteria are satisfied:

• Reduction in proteinuria (expressed as the protein:creatinine ratio in a first morning urine specimen) at 6 months by ≥ 50% of the value at the time of screening, AND

• Estimated GFR (eGFR) at 6 months ≥ 75% of the value at the time of randomization in those with an initial eGFR <75 mL/min/1.73 m^2 ^OR eGFR persistently ≥75 mL/min/1.73 m^2 ^in those whose renal function was ≥75 mL/min/1.73 m^2 ^at the time of randomization.

The proteinuria at the time of screening will be used to determine eligibility and efficacy. While an eGFR ≥40 mL/min/1.73 m^2 ^at screening will be used to determine eligibility, the eGFR at the time of randomization will be used to determine efficacy to account for the hemodynamic impact of intensified combination therapy with ACEi and ARB agents during the Run-In Phase (see below).

The proteinuria endpoint is an objective variable that can be quantitated in a bias-free manner. Although controversy remains over the potential use of proteinuria as a surrogate end point in definitive Phase III clinical trials, proteinuria is an ideal marker for use as an intermediate outcome in earlier phase trials. In particular, proteinuria responds to many therapies within several months, providing an outcome whose response can be assessed in relatively short-term studies [28]. The proteinuria component of the composite was formulated to require a large change (≥50% reduction) to increase the likelihood that changes classified as a positive outcome are clinically relevant and to reduce the influence of small perturbations as might be induced by minor hemodynamic changes. However, due to concerns that reductions in proteinuria may be the result of steadily declining kidney function rather than improvement in glomerular permselectivity, stabilization of eGFR is included in a composite primary endpoint. eGFR will be calculated using the Schwartz equation in patients ≤18 years of age at screening or the Cockroft Gault equation adjusted for body surface area in patients older than 18 years of age, in accord with procedures adopted in the FONT Phase I studies [29-31].

The composite primary outcome will be assessed in all patients enrolled in the trial and who are randomized to one of the three study treatments. In a subgroup analysis, the primary endpoint will be evaluated in the patients in the three experimental groups whose Palb at the time of enrollment exceeds 0.5.

### Secondary Endpoints

Key secondary renal endpoints will include the percent change in the first morning urine protein creatinine ratio from baseline to 6 months, the change in eGFR from baseline to 6 months, both evaluated as continuous variables, and time to a 50% reduction in eGFR and/or ESKD. Patient status [32], and quality of life will be assessed by age-appropriate QOL surveys and the Patient-Reported Outcomes Measurement Information System (PROMIS) score. The adverse effect profile will be determined by clinical evaluation and standard laboratory testing.

### Study Population

Inclusion and exclusion criteria for enrollment are defined in Table 1. The eligibility criterion for steroid resistance or intolerance is defined in recognition of a reluctance of nephrologists to routinely administer a prescribed course of corticosteroids to all patients because of the potential for serious adverse events. FSGS can occur as a consequence of genetic mutations in structural proteins in the podocyte [33-36]. Thus, confirmation of a disease-causing mutation in a podocyte protein is an eligibility criterion for the FONT II trial in patients who lack biopsy-confirmation of the diagnosis of FSGS. Although there is a lack of data on expected response rates to antifibrotic therapy in the presence or absence of podocyte mutations, progressive renal fibrosis is an intrinsic feature of primary FSGS, regardless of whether or not there is a defined genetic mutation. In any event, DNA specimens for storage in the NIDDK Biorepository will be obtained from all patients who enroll in the trial and consent to the procedure. There is no exclusion criterion based on obesity because antifibrotic agents should also be beneficial in patients with obesity-related FSGS.

**Table 1:** FONT II Study Eligibility Criteria

Inclusion Criteria
1. Primary FSGS confirmed by renal biopsy

2. Failure to respond to prior therapy with at least one of the following immunosuppressive medications -- cyclosporine, tacrolimus, mycophenolate mofetil, sirolimus - or other agents prescribed to lower proteinuria

3. Age 1-50 years at onset of proteinuria

4. Age 1-51 years at time of randomization

5. Estimated GFR ≥40 mL/min/1.73 m^2 ^using Schwartz (age <18 yr) or Cockcroft-Gault (age ≥ 18 yr) formula at screening and ≥30 mL/min/1.73 m^2 ^at the end of the Run-In Period and prior to randomization

6. Up/c > 1.0 g protein/g creatinine on first morning void

7. Steroid resistance defined as failure to achieve sustained Up/c < 1.0 following a standard course of prednisone/prednisolone/methylprednisolone prescribed for FSGS therapy, OR contraindication/anticipated intolerance to steroid therapy defined as severe obesity, documented decreased bone density, family history of diabetes, or a psychiatric disorder.

8. Willingness to follow the protocol, including medications, baseline and follow-up visits, and procedures.



**Exclusion Criteria**

1. Lactation, pregnancy, or refusal of birth control in women of child-bearing potential

2. Participation in another therapeutic trial involving protocol mandated administration of a immunosuppressive medication concurrently or 30 days prior to randomization

3. Active/serious infection (including, but not limited to Hepatitis B or C, HIV)

4. History of malignancy

5. Abnormality in age appropriate cancer screening in accord with ACS 2003 guidelines

6. Patients with uncontrolled blood pressure > 140/90 or > 95th percentile for age/height at the end of the run in period

7. Diabetes mellitus, type 1 or 2

8. Organ or bone marrow transplantation

9. Congestive heart failure

10. History of myocardial infarction

11. SLE or multiple sclerosis

12. Hepatic disease defined as serum ALT/AST more than 2.5× the upper limit of normal

13. Hematocrit <27 vol%

14. Immunosuppressive therapy with cyclosporine, tacrolimus, mycophenolate mofetil, azathioprine, rapamycin, or cyclophosphamide in the 30 days prior or Rituximab in the 90 days prior to randomization

15. Use of corticosteroids in the last 30 days except for minimal dosage required for stabilization of edema

16. Prior treatment with the study medications, galactose or adalimumab

17. Allergy to one of the study medications, i.e., adalimumab, galactose, lisinopril, losartan, atorvastatin

18. Abnormal Pap smear (more than carcinoma in situ 1) unless treated and follow-up indicates a normal Pap smear

### Screening and Run In Phase

**Figure 1:**
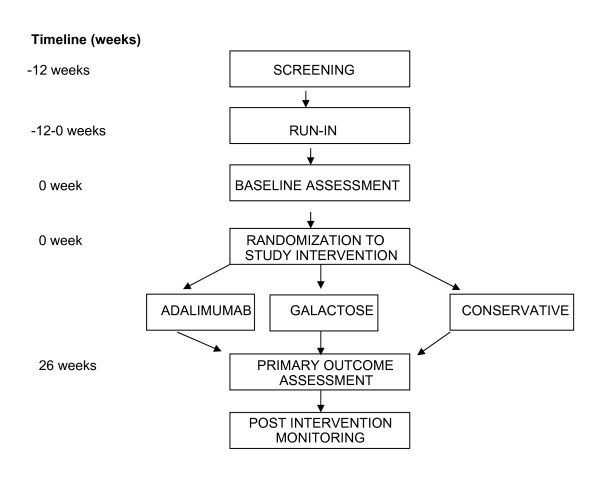
**FONT II Trial Design**.

In order to achieve a comparable baseline assessment of proteinuria and GFR prior to initiation of one of the novel therapies, the patients will be taken off all immunosuppressive medications including corticosteroids (except for a minimal daily or alternate-day dosage to control edema if clinically indicated) for 30 days. In addition, patients will be placed on the maximal tolerated doses of lisinopril, losartan or atorvastatin, based on measurements of blood pressure, serum potassium, creatinine, and cholesterol concentrations. Patients must be on stable doses of the ACEi/ARB treatment for a minimum of 2 weeks prior to randomization into the FONT Phase II study to insure that the initiation of novel therapy does not coincide with a hemodynamically induced change in proteinuria. In order to fully implement the conservative medical therapy regimen in a timely manner, a maximum 12 week Screening/Run-In period will precede randomization (Figure 1).

### Study Medications

Standard conservative medical therapy: All patients will receive a combination of the following three agents: 1) lisinopril: 2) losartan; and 3) atorvastatin. The maximum doses for patients weighing <40 kg will be: lisinopril 10 mg, losartan 25 mg, and atorvastatin 10 mg. For patients weighing >40 kg, the maximal doses will be: lisinopril 20 mg, losartan 50 mg, and atorvastatin 20 mg. Stable dosages of the ACEi and ARB must be achieved by the end of the Screening/Run-in period (2-12 weeks) and remain unchanged for the duration of the 6 month treatment period barring any clinical or laboratory side effects.

The following novel therapies will be administered for 6 months before assessing efficacy;

Adalimumab (Humira^®^): *TNF-α antibody*: The therapeutic dose of adalimumab will be 24 mg/m^2 ^(maximum dose: 40 mg) every other week as a subcutaneous injection for the entire treatment period. Although the pharmacokinetics (PK) data from the FONT Phase I Study indicated enhanced clearance of adalimumab in patients with FSGS and nephrotic-range proteinuria, the dose will not be increased above the standard amount in order to minimize the risk of adverse events [30,31].

Galactose: *sugar*: Galactose (Ferro Pfanstiehl, Waukegan, IL), 0.2 g/kg per dose (maximum dose: 15 g) will be administered orally twice a day. The sugar will be dissolved in 15-30 ml of water and the liquid will be ingested 15-30 minutes before breakfast and dinner.

### Study Protocol

Figure 1 outlines the course of the Phase II trial after completion of the Screening/Baseline period and receipt of study drugs at week 0. Patients will be evaluated after 2, 8, 16, and 26 weeks of the assigned experimental treatment. A follow-up evaluation will be performed at 1 month, 3 months, and 6 months after discontinuation of the novel therapy, and then every 6 months until the end of the funding period.

### Study Mandated Stop Points

• > 50% decline from baseline eGFR and <60 mL/min/1.73 m^2 ^OR a final level <20 mL/min/1.73 m^2^

• ESKD, i.e., initiation of dialysis or receipt of a renal transplant

• Serious Adverse Event (SAE), i.e. grade 4 Common Toxicity Criteria (CTC) toxicity

• Increase in ALT/AST to >2.5× the upper limit of normal

• Onset of congestive heart failure or a myocardial infarction

• Clinical onset of SLE and/or positive ANA ≥1:160

• Serious infection/sepsis

• Malignancy

• Pregnancy

### Statistical Considerations, Efficacy and Power

A hybrid Phase II design as described by Liu et al [37] will be used which incorporates a ranking/selection comparison [38,39] between the treatment groups as well as a minimum activity requirement within each treatment group. FONT II will include a sample size of 42 patients in each of the three treatment arms, with a total sample size of 126 patients. Up to 53 patients may be enrolled in the galactose arm (137 total subjects) to ensure a subgroup of 42 with a P_alb _> 0.5. Although the galactose arm was added after initiation of the study, patient enrollment was very low prior to this change. Therefore, introduction of galactose and removal of rosiglitazone are not anticipated to influence the study population. We next describe the assessment of the minimum activity requirement and the ranking/selection components of the study.

Minimum efficacy component: A two-stage procedure will be conducted to determine if the individual novel experimental treatments achieve a minimal level of efficacy that would signify that the therapies are sufficiently promising to warrant further investigation. In the first stage, patients will be enrolled until each of the 3 treatment groups has accumulated 17 randomized subjects. At this point, randomization will be temporarily halted, and there will be a 6 month pause during which the first set of patients assigned to each of the 3 arms will be allowed to complete the treatment period and have their response to the therapy evaluated. If in either of the experimental groups - adalimumab or galactose groups-- there are one or fewer responders among the first 17 randomized patients, that group will be dropped from further consideration. After the 6-month pause, the study will resume randomization until 42 patients (up to 53 patients may be enrolled in the galactose arm to ensure a subgroup of 42 with a P_alb _> 0.5) are accrued for each agent that passed the initial efficacy threshold.

This component of the design is based on the parameters p0, the estimated response rate to an "ineffective novel therapy", and p1, the projected response rate to an "effective novel therapy" that we would like to evaluate in a RCT. Based on experience gathered from the Glomerular Disease Collaborative Network (University of North Carolina, Chapel Hill, NC), we anticipate that optimal conservative medical therapy will result in response rate (≥50% reduction in proteinuria) of approximately 10%. Taking p0 = 10% and p1 = 30% (designating a 20% improved response for a successful novel therapy), the two-stage procedure described above has Type I and Type II error rates of 2% and 10%, respectively. Any treatment with at least 9 responses (out of 42 patients) will be identified as having a response rate sufficiently greater than 10% and considered to be an "active" agent worthy of further study in subsequent Phase III RCT.

The requirement of 2 or more responses for the two experimental treatments in the first stage reduces the risk of continuing to enroll patients into a treatment group for which the early results indicate a low response rate. On the other hand, the 2-stage design has at least a 98% probability of proceeding to the second stage if the true response rate of either of the two initial experimental treatments is 30% or greater. The overall type I and II error rates of 2 and 10% indicate that for each treatment group, the proposed design has a probability of 2% of incorrectly designating a regimen with a 10% response rate as "active", and a probability of 90% of correctly designating a regimen with a 30% response rate as "active".

### Ranking Selection Component

The ranking/selection component of the design compares the response rates between the treatment groups, and selects the treatment regimen with the best response rate, irrespective of how large or small the advantage over the others may be. The sample size in such a selection design is selected to assure that if one of the treatments has an underlying response rate which is clearly superior to that of the other treatments; it will be selected with high probability. FONT II includes a sample size of 42 (up to 53 patients may be enrolled in the galactose arm to ensure a subgroup of 42 with a P_alb _> 0.5) patients in each of the 3 treatment groups for a total sample size of 126. This will insure that the treatment with the best response rate will be correctly selected with a probability of 85% if one of the treatments has a response rate ≥40% and all the remaining treatments have response rates no higher than 25%.

### Subgroup Analysis

It is anticipated that approximately 80% of the enrolled patients will have P_alb _> 0.5. This value represents the lower limit for increased levels of the permeability factor, which is the putative target of galactose treatment. Therefore, a secondary analysis will be performed in which the response rate of the Galactose arm will be computed separately for the subgroup with P_alb _> 0.5. The ranking-selection comparison of all three groups will also be repeated in patients with P_alb _> 0.5.

### Ethics of Human Subject Research

The use of the full array of experimental therapies in the FONT Phase II Trial has been authorized by the FDA (IND # 100,037). The study protocol, design and consent forms have been approved by Institutional Review Board (IRB) at the two clinical coordinating centers - Cohen Children's Medical Center of New York and University of Michigan-CS Mott Children's Hospital - and 13 other participating sites (Nationwide Children's Hospital, Boston Children's Hospital, Oregon Health Sciences Center, Miami Children's Hospital, Carolinas Medical Center, Stollery Children's Hospital, Medical University of South Carolina, Emory University, Texas Tech University Health Sciences Center, Children's Mercy Hospital, University of Kansas, Mayo Clinic, and Cincinnati Children's Medical Center). Specific study elated information will be made available to the Food and Drug Administration (FDA) and the National Institutes of Health (NIH). All procedures will be in compliance with Health Information Portability and Accountability Act (HIPAA) regulations. The trial is funded primarily by the National Institutes of Health-NIDDK (DK70341) with a small supplemental grant from the NephCure Foundation. The project is listed at ClinicalTrials.gov with the identifier NCT00814255.

## Discussion

FSGS is a clinical entity with a distinctive histopathological appearance, which is either primary or secondary to other etiologies. This lesion accounts for 10-20% of cases of primary nephrotic syndrome in children and up to 35% of cases in adults. In the majority of patients, the primary lesion is refractory to therapeutic interventions with immunosuppressive agents. The final common pathway for advancing disease in primary FSGS is progressive fibrosis leading to ESKD, which occurs in 50 to 75% of patients over a 10-year period [1,2,40-42]. The morbidity and mortality of patients with FSGS is compounded by that of superimposed ESRD. The life expectancy for a 10-year old child who is dialysis-dependent is reduced by approximately 30 to 50 years from the current 84 years in the general US population. Following renal transplantation for FSGS, a high recurrence rate results in a 20-25% allograft loss and further diminishes the likelihood of long-term graft and patient survival. Thus, the longevity of patient survival is directly linked to success in treating FSGS and prolonging native kidney survival [43-45].

The lack of adequate RCTs has hindered clinical research in the treatment of FSGS. The majority of studies that have evaluated potential therapies for FSGS have either been uncontrolled, had poorly defined end-points, or had treatment periods of insufficient length [1,46]. This has engendered a great deal of controversy about the optimal treatment of FSGS and variability in practice from the onset of disease and throughout its course. Cyclosporine is the only drug that has been evaluated and demonstrated by RCT to be useful in pediatric and adult patients [1,2]. The lack of effective agents if cyclosporine fails is underscored by the observation that only 1 out of 14 invited articles dealing with FSGS described alternative treatments, most of which had only been studied in small uncontrolled patient cohorts [47].

This report describes an RCT whose objective is to evaluate potential efficacy of novel therapies that target reduction in renal fibrosis as their main mechanism of action. While this may be an off-target mechanism relative to the approved indication for the test agents, reduction in fibrosis is primary effect in the context of FSGS. However, a major challenge for this type of trial is the development of a dynamic study design aimed at efficiently identifying novel therapeutic approaches and on avoiding large expenditures on unworthy and untested therapeutic agents for serious diseases. The novelty of this project centers on the application of a distinct two step analysis of outcomes to expedite the identification of agents suitable for Phase III testing in patients with a rare glomerular disease, namely treatment-resistant FSGS.

Rosiglitazone was evaluated in the FONT I project [29] and was originally included as a treatment arm in the FONT II trial based on ample preclinical evidence that this drug has antifibrotic properties. However, recent meta-analyses of clinical trials in adults with type 2 diabetes suggest that treatment with thiazolidinediones is associated with an increased risk of myocardial infarction and possibly with overall cardiovascular morbidity and mortality [48,49]. It is unclear if this adverse effect is unique to rosiglitazone or is a class effect that also impacts on the use of the related agent pioglitazone. Although patients with primary FSGS differ from those with type 2 diabetes making it difficult to generalize the risk of serious adverse events from the latter to the former group, concerns about the feasibility of timely recruitment into the FONT Phase II trial forced abandonment of the rosiglitazone arm, leaving a 3-arm study that compares conservative medical therapy, adalimumab, and galactose. The study incorporates comprehensive surveillance for cancer detection because of the increase risk of malignancy in patients treated with TNF-α antagonists [50].

Because of the low incidence and prevalence of patients with FSGS, an efficient and productive study design that fosters collaboration is essential to implement cost-effective trials and to identify and recruit clinical subjects in a timely manner. While conducting Phase I studies, the FONT consortium developed a functional infrastructure that will sustain collaboration. This should hasten the successful performance of studies seeking to develop new therapeutic interventions that will improve the care and outcome of patients with FSGS. The support of the NephCure Foundation, which is dedicated to advancing research into the pathogenesis and treatment of FSGS, has facilitated the creation of the cooperative network and fostered awareness and enthusiasm for the study among patients and their families.

It is anticipated that the proposed clinical trial methodology will overcome existing difficulties involved in the study of rare kidney diseases by implementing cost-effective approaches aimed at identifying novel therapeutic approaches and by avoiding large expenditures in the evaluation of unworthy and/or unsafe therapeutic agents for serious glomerular disorders like FSGS.

## Competing interests

The authors declare that they have no competing interests.

## Authors' contributions

HT and DG conceived and designed the FONT trial and supervised the writing of the manuscript. LW wrote the initial draft of the manuscript. MJ, JG, and TG assisted in the statistical methods aspect of the trial design. SV, VS, MS, and MP assisted in drafting the clinical aspects of the protocol. HT, SV, DG, LW, VS, MS, and MP will be responsible for the clinical conduct of the FONT trial. All of the authors read and approved the final version of the manuscript.

## Pre-publication history

The pre-publication history for this paper can be accessed here:

http://www.biomedcentral.com/1471-2369/12/8/prepub

## References

[B1] Burgess E Management of focal segmental glomerulosclerosis: Evidence-based recommendations Kidney Int 1999 55 S26 S32 10.1046/j.1523-1755.1999.07004.x 10369192

[B2] Benchimol C Focal segmental glomerulosclerosis: pathogenesis and treatment Curr Opin Pediatr 2003 15 171 80 10.1097/00008480-200304000-00006 12640274

[B3] Jafar TH Stark PC Schmid CH for the AIPRD Study Group Angiotensin-converting enzyme inhibition and progression of renal disease. Proteinuria as a modifiable risk factor for the progression of non-diabetic renal disease Kidney Int 2001 60 1131 40 10.1046/j.1523-1755.2001.0600031131.x 11532109

[B4] Gansevoort RT Sluiter WJ Hemmelder MH de Zeeuw D de Jong PE Antiproteinuric effect of blood-pressure-lowering agents: a meta-analysis of comparative trials Nephrol Dial Transplant 1995 10 1963 74 8643149

[B5] Lama G Luongo I Piscitelli A Salsano ME Enalapril: Antiproteinuric effect in children with nephrotic syndrome Clin Nephrol 2000 53 432 6 10879662

[B6] McLaughlin K Jardine AG Angiotensin converting enzyme inhibitors and angiotensin receptor (AT1) antagonists: Either or both for primary renal disease? Nephrol Dial Transplant 1999 14 25 8 10.1093/ndt/14.1.25 10052468

[B7] Milliner DS Morgenstern BZ Angiotensin converting enzyme inhibitors for reduction of proteinuria in children with steroid-resistant nephrotic syndrome Pediatr Nephrol 1991 5 587 90 10.1007/BF00856646 1911144

[B8] Navis G de Zeeuw D de John PE ACE-inhibitors: Panacea for progressive renal disease? Lancet 1997 349 1852 3 10.1016/S0140-6736(97)22026-X 9217751

[B9] Proesmans W Van Wambeke I Van Dyck M Long-term therapy with enalapril in patients with nephrotic-range proteinuria Pediatr Nephrol 1996 10 587 9 10.1007/s004670050166 8897561

[B10] Stiles KP Abbott KC Welch PG Yuan CM Effects of angiotensin-converting enzyme inhibitor and steroid therapy on proteinuria in FSGS: A retrospective study in a single clinic Clin Nephrol 2001 56 89 95 11522100

[B11] Russo D Minutolo R Pisani A Esposito R Signoriello G Andreucci M Balletta MM Coadministration of losartan and enalapril exerts additive antiproteinuric effect in IgA nephropathy Am J Kidney Dis 2001 38 18 25 10.1053/ajkd.2001.25176 11431176

[B12] Woo KT Lau YK Wong KS Chiang GS ACEi/ATRA therapy decreases proteinuria by improving glomerular permselectivity in IgA nephritis Kidney Int 2000 58 2485 91 10.1046/j.1523-1755.2000.00432.x 11115082

[B13] Fried L Orchard TJ Kasiske BL for the Lipids and renal Disease Progression Meta-Analysis Study Group effect of lipoid reduction on progression of renal disease: A meta-analysis Kidney Int 2001 59 260 9 10.1046/j.1523-1755.2001.00487.x 11135079

[B14] Schieppati A Remuzzi G the future of renoprotection: frustration and promises Kidney Int 2003 63 1947 55 10.1046/j.1523-1755.2003.00340.x 14633117

[B15] Brenner BM Retarding the progression of renal disease Kidney Int 2003 63 370 8 10.1046/j.1523-1755.2003.t01-2-00052.x 12787440

[B16] Zoja C Corna D Camozzi D Cattaneo D How to fully protect the kidney in a more severe model of progressive nephropathy: a multidrug approach J Am Soc Nephrol 2002 13 2898 908 10.1097/01.ASN.0000034912.55186.EC 12444208

[B17] Hebert LA Wilmer WA Falkenhain ME Ladson-Wofford SE Nahman S Rovin BH Renoprotection: One or many therapies? Kidney Int 2001 59 1211 1226 10.1046/j.1523-1755.2001.0590041211.x 11260381

[B18] McCarthy ET Sharma R Sharma M Li JZ Ge XL Dileepan KN Savin VJ TNF-alpha increases albumin permeability of isolated rat glomeruli through the generation of superoxide J Am Soc Nephrol 1998 9 433 8 9513905 10.1681/ASN.V93433

[B19] Gertzberg N Neumann P Rizzo V Johnson A NAD(P)H oxidase mediates the endothelial barrier dysfunction induced by TNF-α Am J Physiology 2004 286 L37 L48 10.1152/ajplung.00116.2003 12807699

[B20] Wright SC Zheng H Zhong J Torti FM Larrick JW Role of protein phosphorylation in TNF-induced apoptosis: phosphatase inhibitors synergize with TNF to activate DNA fragmentation in normal as well as TNF-resistant U937 variants J Cell Biochem 1993 53 222 33 10.1002/jcb.240530307 8263039

[B21] Gaur U Aggerwal BB Regulation of proliferation, survival, and apoptosis by members of the TNF superfamily Biochem Pharmacol 2003 66 1403 8 10.1016/S0006-2952(03)00490-8 14555214

[B22] Ortiz A Bustos C Alonso J Alcazar R Lopez-Armada MJ Plaza JJ Gonzalez E Egido J Involvement of tumor necrosis factor-alpha in the pathogenesis of experimental and human glomerulonephritis Adv Nephrol Necker Hosp 1995 24 53 77 7572422

[B23] Cattran D Neogi T McCarthy ET Savin VJ Sharma R Serial estimates of serum permeability activity and clinical correlates in patients with native kidney focal segmental glomerulosclerosis J Am Soc Nephrol 2003 4 448 53 10.1097/01.ASN.0000046960.57614.17 12538746

[B24] Savin VJ Sharma R Sharma M Circulating factor increasing glomerular permeability in recurrent focal segmental glomerulosclerosis N Engl J Med 1996 334 878 883 10.1056/NEJM199604043341402 8596570

[B25] Savin VJ McCarthy ET Sharma R Sharma M Galactose binds to focal segmental glomerulosclerosis permeability factor and inhibits its activity Translational Research 2008 151 288 292 10.1016/j.trsl.2008.04.001 18514139

[B26] DeSmet E Rioux JP Ammann H Deziel C Querin S FSGS permeability factor-associated nephrotic syndrome: remission after oral galactose therapy Nephrol Dial Transplant 2009 24 2938 40 10.1093/ndt/gfp278 19509024

[B27] Meyrier AY Treatment of focal segmental glomerulosclerosis with immunophilin modulation: when did we stop thinking about pathogenesis? Kidney Int 76 487 491 10.1038/ki.2009.204 19494796

[B28] Cattran DC Appel GB Hebert LA A randomized trial of cyclosporine in patients with steroid-resistant focal segmental glomerulosclerosis. North America Nephrotic Syndrome Study Group Kidney Int 1999 56 2220 6 10.1046/j.1523-1755.1999.00778.x 10594798

[B29] Joy MS Gipson DS Dike M Powell L Thompson A Vento S Eddy A Fogo AB Kopp JB Cattran D Trachtman H Phase I trial of rosiglitazone in FSGS: I. Report of the FONT Study Group Clin J Am Soc Nephrol 2009 4 1 39 47 Epub 2008 Dec 10 10.2215/CJN.02310508 19073787 PMC2615712

[B30] Joy MS Gipson DS Powell L MacHardy J Jennette JC Vento S Pan C Savin V eddy A Fogo AB Kopp JB Cattran D Trachtman H Phase I trial of adalimumab in focal segmental glomerulosclerosis: II. Report of the FONT (novel therapies for resistant FSGS) study group Am J Kid Dis 2010 55 50 60 10.1053/j.ajkd.2009.08.019 19932542 PMC2804955

[B31] Peyser A MacHardy N Tarapore F MacHardy J Powell L Gipson DS Savin V Pan C Kump T Vento S Trachtman H Follow-up of phase I trial of adalimumab and rosiglitazone in FSGS: III. Report of the FONT study group BMC Nephrol 2010 11 2 10.1186/1471-2369-11-2 20113498 PMC2823728

[B32] Atkinson MJ Sinha A Hass SL Colman SS Kumar RN Brod M Rowland CR Validation of a general measure of treatment satisfaction, the Treatment Satisfaction Questionnaire for Medication (TSQM), using a national panel study of chronic disease Health Qual Life Outcomes 2004 2 12 10.1186/1477-7525-2-12 14987333 PMC398419

[B33] Caridi G Bertelli R Carrea A Prevalence, genetics and clinical features of patients carrying podocin mutations in steroid resistant nonfamilial focal segmental glomerulosclerosis J Am Soc Nephrol 2001 12 2742 6 11729243 10.1681/ASN.V12122742

[B34] Pollak MR The genetic basis of FSGS and steroid-resistant nephrosis Sem Nephrol 2003 23 141 146 10.1053/snep.2003.50014 12704574

[B35] Ruf RG Lichtenberger A Karle SM Patients with mutations in NPHS2 (podocin) do not respond to standard steroid treatment of nephritic syndrome J Am Soc Nephrol 2004 15 722 32 10.1097/01.ASN.0000113552.59155.72 14978175

[B36] Caridi G Bertelli R DiDuca M Broadening the spectrum of diseases related to podocin mutations J Am Soc Nephrol 2003 14 1278 86 10.1097/01.ASN.0000060578.79050.E0 12707396

[B37] Liu P John Crowley Phase II Selection Designs Handbook of Statistics in Clinical Oncology 2001 Chapter 6 Marcel Dekker 119 127

[B38] Simon R Wittes R Ellenberg Randomized phase II clinical trials Cancer Treatment Rep 1985 69 1375 81 4075313

[B39] Buyse M Randomized designs for early trials of new cancer treatments - An overview Drug Information Journal 2000 34 387 396

[B40] Korbet SM Angiotensin antagonists and steroids in the treatment of focal segmental glomerulosclerosis Semin Nephrol 2003 23 219 28 10.1053/snep.2003.50020 12704582

[B41] Franceschini N Hogan SL Falk RJ Primum non nocere: Should adults with idiopathic FSGS receive steroids? Semin Nephrol 2003 23 229 33 10.1053/snep.2003.50021 12704583

[B42] Cattran DC Rao P Long-term outcome in children and adults with classic focal segmental glomerulosclerosis Am J Kid Dis 1998 32 72 9 10.1053/ajkd.1998.v32.pm9669427 9669427

[B43] Tejani A Stablein DH Recurrence of focal segmental glomerulosclerosis posttransplantation: a special report of the North American Pediatric Renal Transplant Cooperative Study J Am Soc Nephrol 1992 2 12 Suppl S258 S263 1498285 10.1681/ASN.V212s258

[B44] Senggutuvan P Cameron JS Hartley RB Recurrence of focal segmental glomerulosclerosis in transplanted kidneys: analysis of incidence and risk factors in 59 allografts Pediatr Nephrol 1990 4 21 8 10.1007/BF00858431 2206875

[B45] Huang K Ferris ME Andreoni KA Gipson DS The differential effect of race among pediatric kidney transplant recipients with focal segmental glomerulosclerosis Am J Kidney Dis 2004 43 1082 1090 10.1053/j.ajkd.2004.03.017 15168389

[B46] Korbet SM Treatment of primary focal segmental glomerulosclerosis Kidney Int 2002 62 2301 10 10.1046/j.1523-1755.2002.00674.x 12427162

[B47] Ponticelli C Passerini P Other immunosuppressive agents for focal segmental glomerulosclerosis Semin Nephrol 2003 23 242 8 10.1053/snep.2003.50023 12704585

[B48] Selvin E Bolen S Yeh HC Wiley C Wilson LM Marinopoulos SS Feldman L Vassy J Wilson R Bass EB Brancati FL Cardiovascular outcomes in trials of oral diabetes medications: a systematic review Arch Intern Med 2008 168 19 2070 80 10.1001/archinte.168.19.2070 18955635 PMC2765722

[B49] Phillips PJ Twigg SM Oral hypoglycaemics - a review of the evidence Aust Fam Physician 2010 39 9 651 3 20877770

[B50] Smith RA Cokkinides V Eyre HJ American Cancer Society Guidelines for the Early Detection of Cancer CA Cancer J Clin 2003 53 27 43 10.3322/canjclin.53.1.27 12568442

